# Impaired antibody responses were observed in patients with type 2 diabetes mellitus after receiving the inactivated COVID-19 vaccines

**DOI:** 10.1186/s12985-023-01983-7

**Published:** 2023-02-07

**Authors:** Feng Xiang, Boyu Long, Jiaoxia He, Feifei Cheng, Sijing Zhang, Qing Liu, Zhiwei Chen, Hu Li, Min Chen, Mingli Peng, Wenwei Yin, Dongfang Liu, Hong Ren

**Affiliations:** 1 Key Laboratory of Molecular Biology for Infectious Diseases (Ministry of Education), Institute for Viral Hepatitis, Department of Infectious Diseases, the Second Affiliated Hospital, Chongqing Medical University, Chongqing, China; 2 Department of Endocrine, The Second Affiliated Hospital, Chongqing Medical University, Chongqing, China

**Keywords:** Antibody responses, COVID-19 vaccines, Memory B cells, Safety, Type 2 diabetes mellitus

## Abstract

**Background:**

Patients with type 2 diabetes mellitus (T2DM) have been reported to be more susceptible to 2019 novel coronavirus (2019-nCoV) and more likely to develop severe pneumonia. However, the safety and immunological responses of T2DM patients after receiving the inactivated vaccines are not quite definite. Therefore, we aimed to explore the safety, antibody responses, and B-cell immunity of T2DM patients who were vaccinated with inactivated coronavirus disease 2019 (COVID-19) vaccines.

**Methods:**

Eighty-nine patients with T2DM and 100 healthy controls (HCs) were enrolled, all of whom had received two doses of full-course inactivated vaccines. At 21–105 days after full-course vaccines: first, the safety of the vaccines was assessed by questionnaires; second, the titers of anti-receptor binding domain IgG (anti-RBD-IgG) and neutralizing antibodies (NAbs) were measured; third, we detected the frequency of RBD-specific memory B cells (RBD-specific MBCs) to explore the cellular immunity of T2DM patients.

**Results:**

The overall incidence of adverse events was similar between T2DM patients and HCs, and no serious adverse events were recorded in either group. Compared with HCs, significantly lower titers of anti-RBD-IgG (*p* = 0.004) and NAbs (*p* = 0.013) were observed in T2DM patients. Moreover, the frequency of RBD-specific MBCs was lower in T2DM patients than in HCs (*p* = 0.027). Among the 89 T2DM patients, individuals with lower body mass index (BMI) had higher antibody titers (anti-RBD-IgG: *p* = 0.009; NAbs: *p* = 0.084). Furthermore, we found that sex, BMI, and days after vaccination were correlated with antibody titers.

**Conclusions:**

Inactivated COVID-19 vaccines were safe in patients with T2DM, but the antibody responses and memory B-cell responses were significantly decreased compared to HCs.

***Trial registration number and date*:**

NCT05043246. September 14, 2021. (Clinical Trials.gov)

**Supplementary Information:**

The online version contains supplementary material available at 10.1186/s12985-023-01983-7.

## Background

Coronavirus disease 2019 (COVID-19) is an infectious disease that caused a global epidemic of viral pneumonia in 2019. The virus that causes the disease, SARS-CoV-2, is extremely contagious [1, 2].

Type 2 diabetes mellitus (T2DM) is a common chronic metabolic disease, and the global prevalence of diabetes has continued to increase over the past 50 years. The development of T2DM is primarily caused by a combination of two main factors: the lack of insulin secreted by pancreatic β**-**cells and the diminished ability of insulin-sensitive tissues to respond to insulin [3]. Higher rates of hospitalization and severe pneumonia and mortality have been observed in diabetes mellitus (DM) patients infected with SARS-COV-2 than in nondiabetic patients [4, 5]. The inadequate function of the immune system in patients with uncontrolled DM is one of the reasons [6]. Prior studies have proven that patients with uncontrolled DM have impaired antibody responses following influenza and hepatitis B vaccines [7–9]. Some researches have revealed that immunological responses in patients with DM did not differ from those in healthy individuals. For example, Sourij et al. [10] reported that patients with both type 1 diabetes mellitus and T2DM who received COVID-19 vaccines showed humoral immune responses similar to those of healthy controls (HCs). Parthymou et al. [11] found that there was no correlation between antibody titers and DM. However, there were some conflicting conclusions. One CAVEAT study proved that T2DM patients with poor glycemic control had weaker immunity after being vaccinated against COVID-19 than patients who were normoglycemic and well-controlled [12]. Another study showed that SARS-CoV-2 BNT162b2 vaccines elicited weak immune responses in DM patients compared with nondiabetic patients [13].

Although nearly 3 years have passed since COVID-19 outbreak, it has yet to be officially declared over. But many countries have announced the resumption of work and production, and individuals with underlying diseases (including T2DM) are very necessary to be vaccinated due to their insufficient immunity. Therefore, it is urgent to explore the safety and immunogenicity of post-vaccination vaccines for this special population. CoronaVac and BBIBP-CorV vaccines are widely used in China, both of which are inactivated vaccines. As mentioned above, the antibody responses of T2DM patients following inactivated vaccines are poorly elucidated. Therefore, this study concentrated on the immune responses (humoral immunity and cellular immunity) of T2DM patients who were vaccinated with inactivated COVID-19 vaccines.

## Methods

### Study design and population

In this study, 89 adult patients with T2DM and 100 adult healthy individuals were enrolled between July 29, 2021, and October 22, 2021, at the Second Affiliated Hospital of Chongqing Medical University. All participants had received a full-course of inactivated COVID-19 vaccines (CoronaVac and BBIBP-CorV). The eligibility criteria were as follows: diagnosed with T2DM prior to vaccination for the patients group; no recorded disease status for HCs; and 21–105 days had passed after the second dose of inactivated vaccines for all participants. The main exclusion criteria were: SARS-CoV-2 infection or a history of suspected clinical SARS-CoV-2 infection before receipt of the first dose of vaccines; malignant tumor; pregnancy; immunosuppressant administration within 6 months; and acute inflammatory diseases. This study followed the guidelines of the Declaration of Helsinki and was approved by the Ethics Committee of the Second Affiliated Hospital of Chongqing Medical University (No. 133). All participants gave their informed consent before their inclusion in the study.

### Safety assessment

Questionnaires were used to record adverse events within 7 days and 30 days after the second dose of vaccines (We did not record adverse events after the first dose of vaccines). The classification of adverse events referred to the classification scale issued by the National Medical Products Administration of China (version 2019).

### Serology assays

The titers of anti-receptor binding domain IgG (anti-RBD-IgG) and neutralizing antibody (NAbs) were detected by capture chemiluminescence immunoassays (CLIA). Samples were evaluated with MAGLUMI 2000 (Snibe, Shenzhen, China). The cutoff values of anti-RBD-IgG and NAbs were 1.0 AU/mL and 0.15 ug/mL, respectively. (The converted log values were used for analysis, and the cutoff values of anti-RBD-IgG and NAbs after conversion were 0 AU/mL and 0 ug/mL, respectively). The antibodies were considered seropositive when the detected values were greater than the corresponding cutoff values; otherwise, they were considered seronegative.

### SARS-COV-2 RBD-specific MBCs detection

Fresh peripheral blood mononuclear cells (PBMCs) were isolated by Ficoll density gradient centrifugation. After that, the PBMCs were stained with multiple fluorescent-coupled antibodies. Finally, the samples were assessed by flow cytometry. Details and strategy of the procedure can be obtained from supplementary materials.

### Statistical analysis

All continuous variables were checked for normality assumption. Median and interquartile range (IQR) were used to describe continuous variables, while frequency or percentage was used to describe categorical variables. Continuous variables were compared using the T-test or Mann–Whitney U test for two groups and Kruskal–Wallis test for three groups (Bonferroni correction was used for the results of multiple comparisons). Categorical variables were compared using the chi-square or Fisher’s exact test; in addition, we used multiple linear regression analysis to obtain factors that may affect antibody titers. Two-tailed *P* values were reported, with *P* < 0.05 indicating statistical significance (*P < 0.05; **P < 0.01; ***P < 0.001). Missing data were not interpolated. Calculations were performed using SPSS 26 software and GraphPad Prism 9.0

## Results

### Population characteristics

This study recruited a total of 189 participants: 89 patients diagnosed with T2DM and 100 adult healthy individuals. There were no statistically significant differences in sex, age, BMI, and days between the two groups. Notably, the median age of participants in both groups was over 60 years old. In the T2DM patients group, 75.3% received the CoronaVac, 23.6% the BBIBP-CorV, and 1.1% the BBIBP-CorV + CoronaVac; in HCs, the percentages were 54.0%, 39.0%, and 7.0%, respectively. The median HbA1c (Hemoglobin A1c) in the T2DM group was 7.4%. In addition, FPG (Fasting Plasma Glucose) in the T2DM group was significantly higher than that in HCs (*p* < 0.001). The red blood cell count, white blood cell count, and lymphocyte count were similar between the two groups, while the platelet count in the T2DM group was lower than that in the HCs group (Table 1).

**Table 1:** Baseline characteristics of the study populations

Variables	T2DM (n = 89)	HCs (n = 100)	*P* value
*Sex*			
Male, n (%)	49 (55.1%)	56 (56.0%)	0.896
Female, n (%)	40 (44.9%)	44 (44.0%)	
*Ages (years)*	62 (53, 70)	61 (54, 66)	0.226
*BMI (kg/m*^*2*^*)*	24.1 (22.1, 26.4)	24.4 (22.3, 26.6)	0.230
*Days after full-course vaccination (days)*	50 (32, 88)	57 (36, 75)	0.534
*Days between the 1st and 2nd doses*	27 (22, 33)	26 (24, 33)	0.646
*COVID-19 vaccines*			
CoronaVac, n (%)	67 (75.3%)	54 (54.0%)	0.004
BBIBP-CorV, n (%)	21 (23.6%)	39 (39.0%)	
BBIBP-CorV + CoronaVac, n (%)	1 (1.1%)	7 (7.0%)	
*HbA1c (%)*	7.4 (6.4, 9.9)	–	–
*FPG (mmol/L)*	7.5 (6.3, 9.4)	5.3 (5.0, 5.9)	0.000
*Laboratory examination*			
Red blood cell count (10^12^/L)	4.6 (4.2, 5.0)	4.7 (4.4, 5.2)	0.166
White blood cell count (10^9^/L)	6.0 (4.7, 7.2)	5.9 (4.8, 6.9)	0.691
Lymphocyte count (10^9^/L)	1.7 (1.3, 2.2)	1.7 (1.5, 2.2)	0.220
Platelet count (10^9^/L)	171 (174, 212)	193 (166, 232)	0.004

Data were presented as median (range) or number (%)

*BMI* body mass index, *HbA1c* Hemoglobin A1c, *FPG* Fasting Plasma Glucose

### Inactivated COVID-19 vaccines were safe in patients with T2DM

The overall incidence of adverse events within 7 days of vaccination was 6.7% in the T2DM group and 6% in the HCs group. Within 30 days of vaccination, the overall incidence of adverse events in the T2DM group and HCs group was 5.6% and 8%, respectively. The differences between the two groups were not statistically significant (7 days: *p* = 0.835; 30 days: *p* = 0.518). Local adverse events mainly manifested as pain, swelling, and itching at the site of vaccination. Furthermore, systemic adverse events were uncommon. In the T2DM patients group, systemic adverse events, including lethargy and cough, were reported in 5 patients. However, only 1 participant developed systemic adverse events in HCs, namely, lethargy. Of note, no grade 3 and 4 adverse events were observed among the 189 participants (Table 2). Briefly, the incidence of adverse events in T2DM patients who were vaccinated with a full-course of CoronaVac or BBIBP-CorV (or both) was similar to that of HCs, with only a few patients who developed mild adverse reactions.

**Table 2:** Adverse events of COVID-19 inactivated vaccines in all participants

Variables	T2DM (n = 89)	HCs (n = 100)	*P* value
*Overall adverse events within 7 days*	6 (6.7%)	6 (6%)	0.835
*Overall adverse events within 30 days*	5 (5.6%)	8 (8%)	0.518
*Local adverse events*			
Swelling	2 (2.2%)	2 (2%)	1.000
Pain	2 (2.2%)	6 (6%)	0.285
Itch	1 (1.1%)	–	0.471
*Systemic adverse events*			1.000
Lethargy	1 (1.1%)	1 (1%)	1.000
Cough	1 (1.1%)	–	0.471
Fatigue	2 (2.2%)	–	0.220
Pruritus	1 (1.1%)	–	0.471
*Grade 3 and 4 adverse events*	–	–	1.000

Data were presented as number (%). The types of unrecorded adverse events were not listed here

### Impaired antibody responses were observed in patients with T2DM

**Fig. 1:**
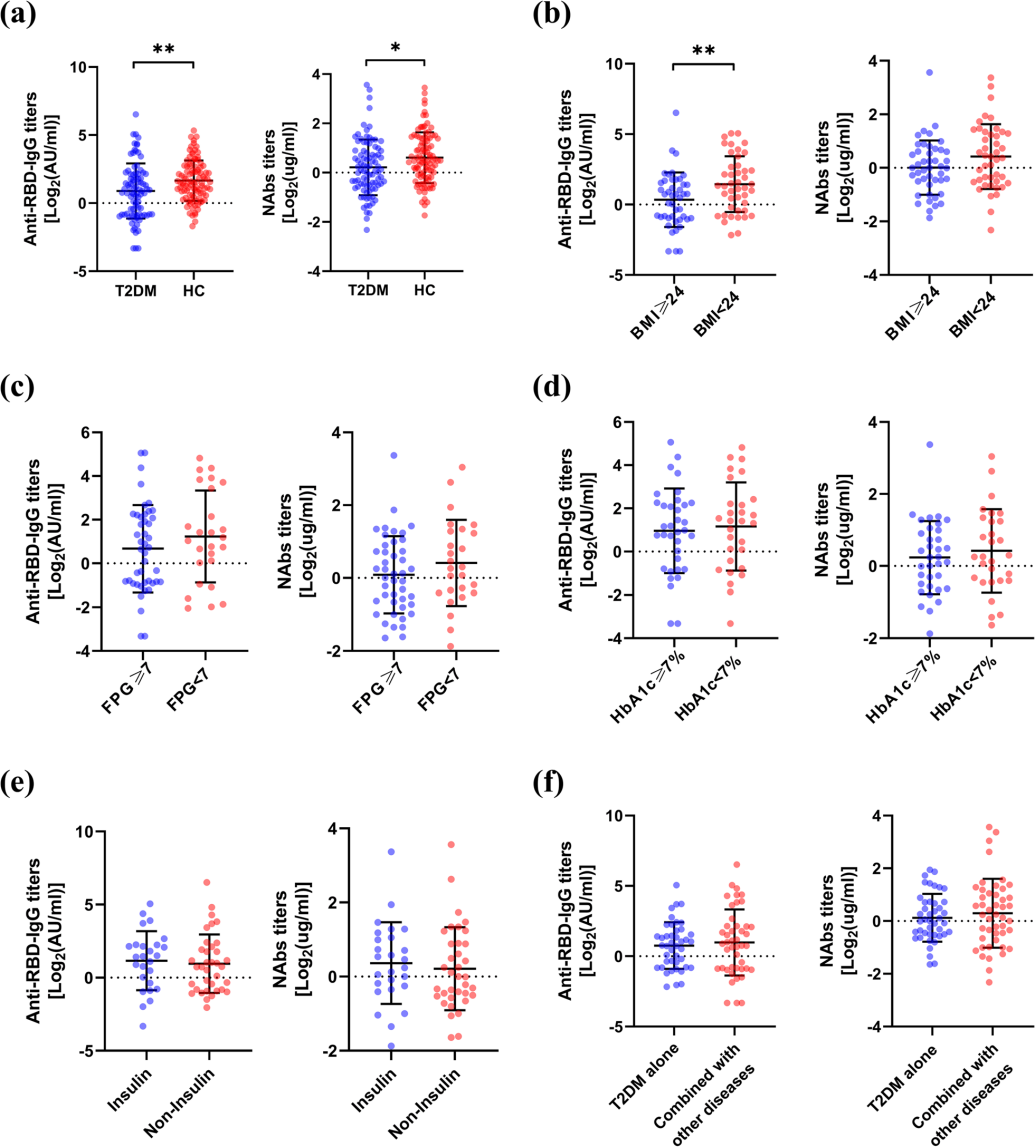
The titers of anti-RBD-IgG and NAbs. **a** All individuals of T2DM and HCs. **b** BMI in patients with T2DM. **c** FPG in patients with T2DM. **d** HbA1c in patients with T2DM. **e** Treatment with and without insulin in patients with T2DM. **f** T2DM alone and T2DM combined with other independent chronic diseases. The horizontal dotted lines represent the cutoff values

Subsequently, we detected the anti-RBD-IgG and NAbs levels of participants after receiving the vaccines. Generally, the titers of anti-RBD-IgG (*p* = 0.004) and NAbs (*p* = 0.013) in T2DM patients (n = 89) were significantly lower than those in HCs (n = 100). The mean antibody titers of anti-RBD-IgG in T2DM patients and HCs were 0.89 and 1.65 log_2_ AU/mL, respectively, and the mean titers of NAbs were 0.24 and 0.61 log_2_ug/mL, respectively (Fig. 1a). The positive rates of both antibodies in T2DM patients were also significantly lower than those in HCs (anti-RBD-IgG: *p* < 0.001; NAbs: *p* = 0.006) (Additional file 1: Fig. S1a) (additional figures are given in Additional file 1). Well-controlled BMI and FPG levels benefit the prognosis of patients with T2DM. Therefore, we compared the antibody titers in T2DM patients with different BMI and FPG levels. Consistent with our expectation, the titers of anti-RBD-IgG in the BMI < 24 group (n = 45) were significantly higher than those in the BMI ≥ 24 group (n = 44, *p* = 0.009). A similar trend was observed in the NAbs titers (*p* = 0.084) (Fig. 1b). Likewise, the positive rates of both antibodies were also higher in T2DM patients with BMI < 24, although the difference was not statistically significant (anti-RBD-IgG: *p* = 0.139; NAbs: *p* = 0.24) (Additional file 1: Fig. S1b). Compared with the FPG ≥ 7 group (n = 45), we found that the antibody titers (anti-RBD-IgG: *p* = 0.242; NAbs: *p* = 0.214) (Fig. 1c) and positive rates (anti-RBD-IgG: *p* = 0.072; NAbs: *p* = 0.303) (Additional file 1: Fig. S1c) were higher in the FPG < 7 group (n = 26), but the differences were not statistically significant.

In addition, we performed a subgroup analysis of HbA1c in the T2DM group, as HbA1c reflects the average plasma glucose levels over the past 2–3 months. We found that the titers of both antibodies (anti-RBD-IgG: *p* = 0.688; NAbs: *p* = 0.495) were similar between the HbA1c ≥ 7% group (n = 35) and the HbA1c < 7% group (n = 29) (Fig. 1d). At the same time, there were no differences in seroprevalence between the two groups (anti-RBD-IgG: *p* = 0.93; NAbs: *p* = 0.69) (Additional file 1: Fig. S1d).

Furthermore, we analyzed the antibody levels of T2DM patients treated with insulin (among the hypoglycemic drugs, only insulin was used) and those whose regimens did not include insulin (their regimens also did not contain other hypoglycemic drugs). The results showed that both anti-RBD-IgG and NAbs titers in the insulin group (n = 26) were higher than those in the non-insulin group (n = 36), despite the differences were not statistically significant (anti-RBD-IgG: *p* = 0.392; NAbs: *p* = 0.602) (Fig. 1e); the trend in positive rates of antibodies was similar to titers (anti-RBD-IgG: *p* = 0.326; NAbs: *p* = 0.265) (Additional file 1: Fig. S1e). To investigate whether coexisting with other independent chronic diseases contributed to inferior antibody titers compared to patients with T2DM alone. We assessed antibody titers in patients who were diagnosed with only T2DM (n = 46) and with other independent chronic diseases (n = 43) (including at least one of the following diseases: primary hypertension, coronary heart disease, chronic hepatitis B, chronic obstructive pulmonary disease, and primary chronic kidney disease). We found no differences in antibody titers (anti-RBD-IgG: *p* = 0.743; NAbs: *p* = 0.844) or positive rates (anti-RBD-IgG: *p* = 0.649; NAbs: *p* = 0.250) between the two groups (Fig. 1f and Additional file 1: Fig. S1f).

In the sex subgroup analysis, no differences were found in antibody titers and positive rates between male (n = 49) and female (n = 40) patients with T2DM. It was worth noting that the antibody titers of males (n = 56) were lower than those of females (n = 44) in HCs (anti-RBD-IgG: *p* = 0.006; NAbs: *p* = 0.007) (Additional file 1: Fig. S2a). The trend in positive rates was similar to that in titers (anti-RBD-IgG: *p* = 0.042; NAbs: *p* = 0.017) (Additional file 1: Fig. S1g). In terms of age, antibody titers and positive rates were also similar between participants older than 65 years and younger than 65 years, both in T2DM patients (≥ 65y, n = 43; < 65y, n = 46) and in HCs (≥ 65y, n = 32; < 65y, n = 68) (Additional file 1: Figs. S1h and S2b). We then sought to explore whether the administration of different inactivated vaccines would result in different responses, and we found that the titers of both antibodies in HCs vaccinated with CoronaVac were higher than those vaccinated with BBIBP-CorV (anti-RBD-IgG: *p* = 0.008; NAbs: *p* = 0.01). In the T2DM group, there were no differences in anti-RBD-IgG antibody titers (*p* = 0.078) or positive rates (*p* = 0.059) between patients who received CoronaVac and those who received BBIBP-CorV, but the NAbs titers (*p* = 0.006) and positive rates (*p* = 0.006) of the former were higher than those of the latter (Additional file 1: Figs. S1i and S2c).

In summary, the antibody responses of T2DM patients after inactivated COVID-19 vaccines were significantly impaired compared to those of healthy individuals.

### B-cell responses were attenuated following inactivated COVID-19 vaccines in T2DM patients

**Fig. 2:**
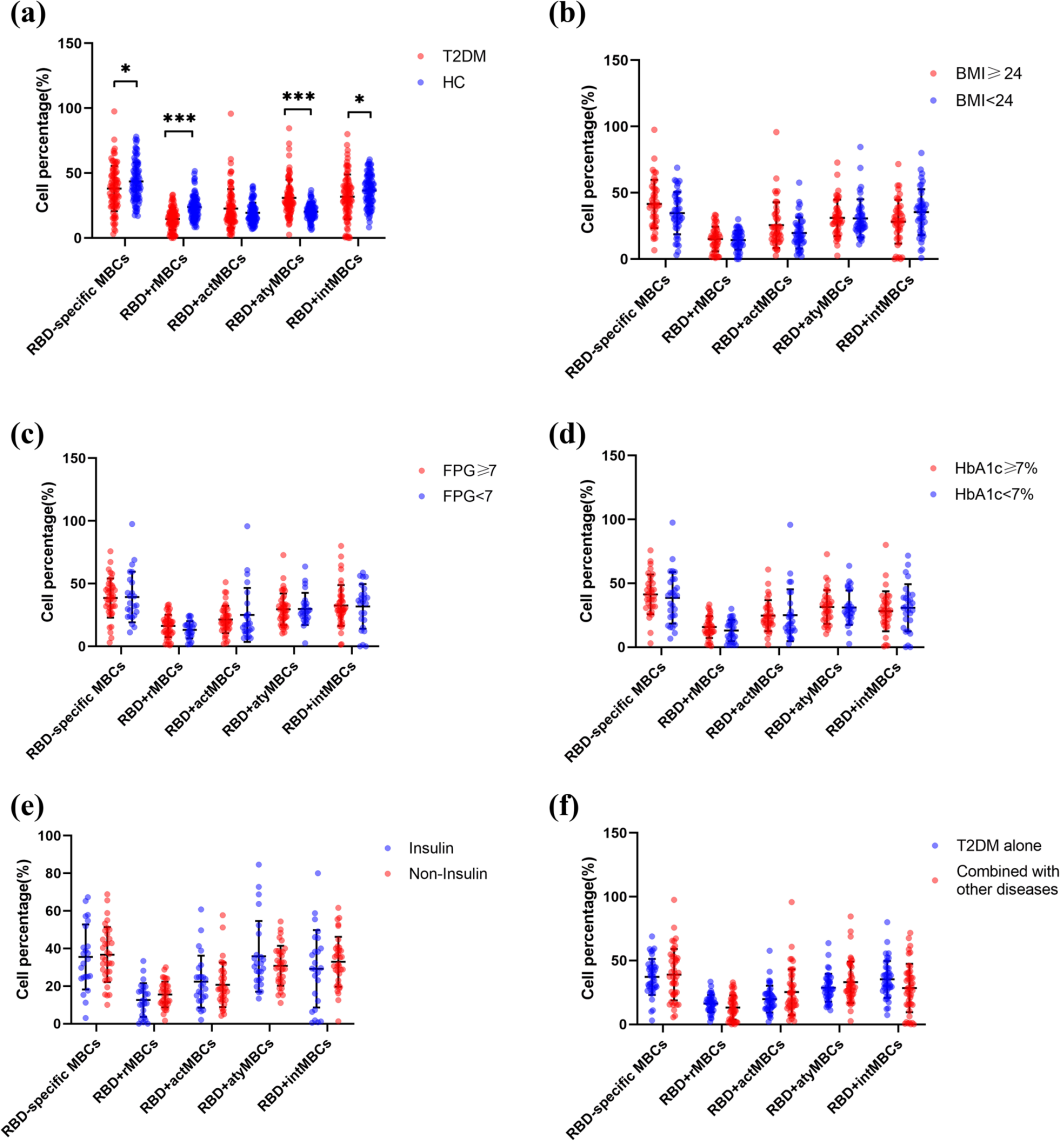
The percentage of RBD-specific MBCs and their subsets. **a** All participants of T2DM and HCs. **b** BMI in patients with T2DM. **c** FPG in patients with T2DM. **d** HbA1c in patients with T2DM. **e** Treatment with and without insulin in patients with T2DM. **f** T2DM alone and T2DM combined with other independent chronic diseases

Memory B cells produce accelerated and stronger immune responses in secondary immune responses. Therefore, the frequency of RBD-specific MBCs and their subsets was analyzed. We discovered that HCs showed a higher percentage of RBD-specific MBCs than T2DM patients (*p* = 0.027). In RBD-specific MBCs subsets, the percentage of RBD-specific resting memory B cells (RBD + rMBCs) (*p* < 0.001) and RBD-specific intermediate memory B cells (RBD + intMBCs) (*p* < 0.035) was also lower in T2DM patients than in HCs, but the trend of RBD-specific atypical memory B cells (RBD + atyMBCs) was reversed (*p* < 0.001) (Fig. 2a). Moreover, the percentage of activated memory B cells (RBD + actMBCs) was higher in the T2DM group, but the differences were not statistically significant (*p* = 0.397). In the T2DM cohort, no statistically significant differences were found in the percentage of RBD-specific MBCs and their subsets, regardless of BMI, FPG, and HbA1c levels (Fig. 2b–d). In terms of medication, treatment with or without insulin appeared to not affect RBD-specific MBCs (Fig. 2e); similar results were revealed between patients diagnosed with only T2DM and those with other independent chronic diseases (Fig. 2f).

Taken together, RBD-specific MBCs responses were attenuated in T2DM patients compared with healthy individuals after inactivated COVID-19 vaccines.

### Decreased antibody titers over time were found in both T2DM patients and HCs

**Fig. 3:**
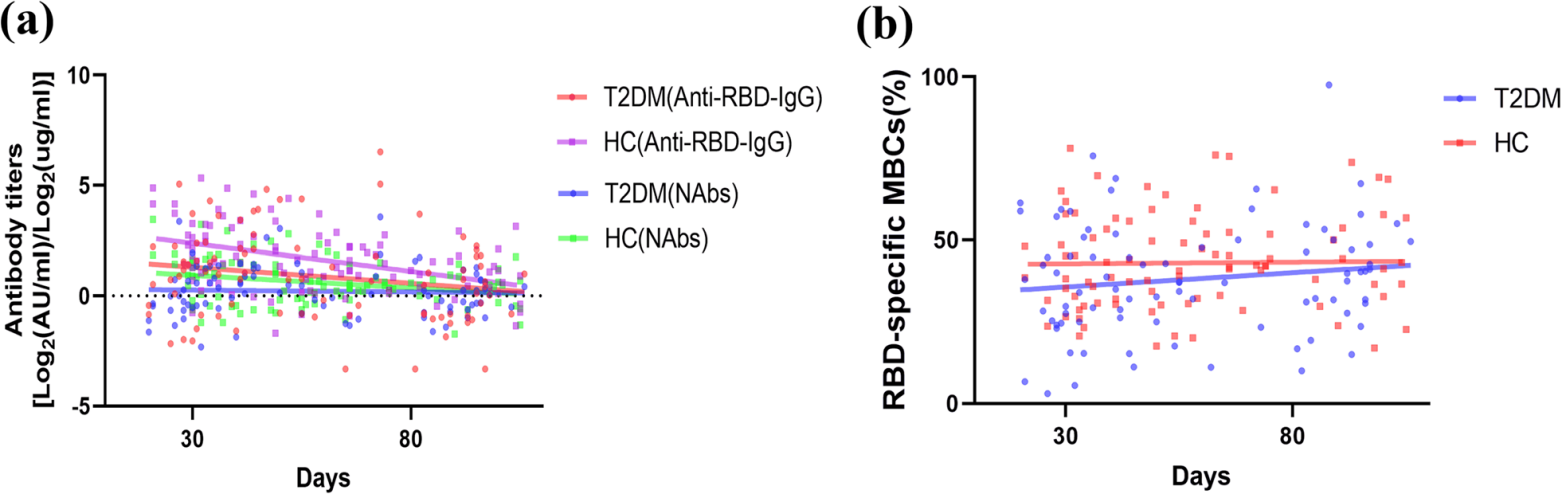
Trend of antibody titers and RBD-specific MBCs over time (21–105 days after vaccination). **a** Anti-RBD-IgG and NAbs titers, **b** the percentage of RBD-specific MBCs

The trend in antibody titers and the frequency of RBD-specific MBCs over time (21–105 days after vaccination) was also analyzed. On the whole, both antibody titers declined as more days passed after receiving the full-course vaccines in all participants (Fig. 3a). Nevertheless, the percentage of RBD-specific MBCs in HCs remained stable, and the percentage of RBD-specific MBCs in T2DM patients showed a slight upward trend (Fig. 3b).

### Sex, BMI, and days were associated with antibody titers

We pooled all T2DM patients to perform multiple linear regression analysis, in which female sex was a positive factor for antibody titers (anti-RBD-IgG: β = 0.608, *p* = 0.001; NAbs: β = 0.501, *p* = 0.014), while BMI was a negative factor for antibody titers (anti-RBD-IgG: β =  − 0.528, *p* = 0.003; NAbs: β =  − 0.475, *p* = 0.021). In addition, the days after full-course vaccination were inversely correlated with anti-RBD-IgG (β =  − 0.419, *p* = 0.013). The interaction terms between antibody titers and the variables (age, FPG, HbA1c, insulin use, and T2DM patients combined with other independent chronic diseases, albumin, and creatinine) were not powered to result in statistically significant *p* values (Tables 3 and 4).

**Table 3:** Multiple linear regression analysis for anti-RBD-IgG in T2DM patients

Variables	β	95% CI	*P* value
Age	− 0.343	(1.000, 0.012)	0.120
Sex (female)	0.608	(1.113, 3.692)	0.001
BMI	− 0.528	(− 0.625, − 0.142)	0.003
FPG	− 0.144	(− 0.375, 0.170)	0.443
HbA1c	− 0.154	(− 0.476, 0.195)	0.393
Insulin (use)	0.032	(− 1.476, 1.731)	0.870
Days after full-course vaccination	− 0.419	(− 0.047, − 0.006)	0.013
Other independent chronic diseases (with)	− 0.075	(− 1.524, 0.961)	0.642
Albumin	0.214	(− 0.023, 0.132)	0.158
Creatinine	0.038	(− 0.005, 0.007)	0.807

*BMI* body mass index, *FPG* Fasting Plasma Glucose, *HbA1c* Hemoglobin A1c, CI confidential interval, *β* standard regression coefficient, *D–W* Durbin–Waston: 1.926

*p* < 0.05 was considered statistically significant

**Table 4:** Multiple linear regression analysis for NAbs in T2DM patients

Variables	β	95% CI	*P* value
Age	− 0.170	(− 0.047, 0.024)	0.509
Sex (female)	0.501	(0.234, 1.872)	0.014
BMI	− 0.475	(− 0.337, − 0.030)	0.021
FPG	− 0.071	(− 0.200, 0.146)	0.751
HbA1c	− 0.122	(− 0.272, 0.154)	0.569
Insulin (use)	− 0.109	(− 1.247, 0.789)	0.645
Days after full-course vaccination	− 0.210	(− 0.020, 0.006)	0.264
Other independent chronic diseases (with)	− 0.106	(− 1.000, 0.577)	0.583
Albumin	0.253	(− 0.015, 0.083)	0.162
Creatinine	− 0.044	(− 0.004, 0.003)	0.814

*BMI* body mass index, *FPG* Fasting Plasma Glucose, *HbA1c* Hemoglobin A1c, *CI* confidential interval, *β* standard regression coefficient, *D–W* Durbin–Waston: 2.055

*p* < 0.05 was considered statistically significant

## Discussion

Since the early pandemic, DM has been certified as a risk factor for poor outcomes in COVID-19 [14]. Both innate and adaptive immunity are compromised in DM patients [15]. Moreover, chronic hyperglycemia can compromise innate and humoral immunity [16]. The current research evidence in this area is limited. Hence, it is highly necessary to investigate the safety and immune responses of patients with T2DM post-vaccination. Our study evaluated the safety of inactivated COVID-19 vaccines in T2DM patients and focused on the antibody titers and the frequency of RBD-specific MBCs. Furthermore, antibody titers of different BMI, FPG, and HbA1c levels in T2DM patients, as well as factors that may influence antibody responses, were also explored.

Herein, we reported an adverse event rate of 6.7% within 7 days after vaccination with inactivated vaccines in T2DM patients and 6% in HCs. This incidence was significantly lower than phase I/II trials of BBIBP-CorV (23–29%) [17] and phase I/II clinical trials of CoronaVac in Chinese populations (19–33%) [18]. The differences could be due to population size and self-report. The most common local adverse events were pain and swelling at the injection site, and a very small number of systemic adverse events, including lethargy and fatigue, occurred. This was consistent with Francesca et al. [19]. It was important to note that no serious adverse events requiring hospitalization have been observed in patients with T2DM. In general, our study provided preliminary evidence that inactivated COVID-19 vaccines were safe for T2DM patients.

Concerning the antibody responses, more than half of T2DM patients developed seropositive transformation of anti-RBD-IgG and NAbs (positive rate: 65% and 53%, respectively), but the positive rates were significantly lower than those of HCs (88% and 72%, respectively), which stayed in step with the report of Nanny et al. [20]. Consistent presentation was also obtained in antibody titers. Furthermore, in comparison to HCs, T2DM patients had a significantly lower percentage of RBD-specific MBCs. Memory B cells could differentiate into plasma cells and produce antibodies after reinoculation with COVID-19 vaccines. Researches have confirmed that T2DM is characterized by chronic inflammation, and this state of chronic inflammation results in an inability to generate adequate immunological responses to specific infections, including COVID-19 [21]. Therefore, we surmised that insufficient production of RBD-specific MBCs and impaired immunity might be the reasons for impaired antibody responses in T2DM patients.

Another finding of our study was that the titers of anti-RBD-IgG and NAbs performed a gradually declining trend over time. However, the frequency of RBD-specific MBCs showed a slight increase within 21–105 days after two doses of inactivated COVID-19 vaccines, and no downward trend was observed. The aforementioned results were not surprising because one study showed that 2 months after the second dose of BNT162b2 vaccines, specific antibody levels declined, but highly specific memory B cells continued to increase [22]. We did not observe a significant increase in the frequency of RBD-specific MBCs. On the one hand, it might be caused by the differences in the characteristics of the populations; on the other hand, our study was cross-sectional, which was not the optimal way to reflect the changes in RBD-specific MBCs. Despite this, we still proved that two doses of inactivated COVID-19 vaccines induced stable levels of RBD-specific MBCs in T2DM patients, and there was no decrease within the time frame of the study. Immune memory is the prerequisite for vaccines to protect the body for a long time. When vaccinated individuals are exposed to SARS-CoV-2, RBD-specific MBCs can rapidly differentiate into plasma cells and produce antigen-clearing antibodies, which is also an intrinsic factor in the effect of the vaccines. In this study, we show that RBD-specific MBCs persist in patients with T2DM for 3 months after vaccination, which may contributed to reducing the rates of hospitalization and mortality of patients with T2DM due to COVID-19.

Regarding subgroup exploration, T2DM patients with higher BMI, FPG, and HbA1c levels developed lower titers of anti-RBD-IgG and NAbs following the inactivated COVID-19 vaccines, although the results of the latter two subgroups were not statistically significant. Our regression analysis suggested a negative correlation between BMI and antibody titers. These suggested that the antibody responses were worse in T2DM patients with higher BMI. Previous studies have revealed that poor vaccine-induced immune responses have been observed in obese individuals for hepatitis B, influenza A/pH1N1, tetanus, and rabies vaccines [23]. The presence of central obesity was associated with a lower antibody concentration following vaccination [24]. It should be noted that the function of T-lymphocytes is defective in poorly controlled patients with DM [25, 26]. The memory CD4 + T-cell responses were negatively correlated with FPG and HbA1c, indicating that the higher the glycemic level was, the more severe the T cells were hypofunction [26]. At the same time, B-cell function was impaired in uncontrolled diabetes because B cells need T cells to activate into antibody-producing plasma cells [27]. Hence, this might explain why the antibody responses to inactivated vaccines were impaired in T2DM patients with poor BMI, FPG, and HbA1c control. Although regression analysis suggested that FPG and HbA1c levels did not affect antibody titers, it is necessary to control the above indicators according to the guidelines in T2DM patients, which is not only conducive to preventing complications but also to improve the immune responses.

Insulin is an important hypoglycemic drug in the treatment of T2DM patients. This research suggested that antibody titers and positive rates of T2DM patients treated with insulin were higher than those treated without insulin, which might be attributed to its good glycemic control effect and anti-inflammatory effect [28–30]. T2DM patients often develop other chronic diseases, especially primary hypertension and coronary heart disease. Hypertension and coronary artery disease are common comorbidities and dangerous factors for infection and serious COVID-19 [31]. Previous studies have shown that hypertension is closely associated with lower antibody titers [24]. We hypothesized that the combination of multiple independent chronic diseases might lead to worse antibody responses than patients diagnosed with T2DM alone, but the results indicated that no differences were observed between the two groups in anti-RBD-IgG and NAbs titers, and this factor was not correlated with antibody titers either. Another point worth noting in our study was that healthy females had significantly higher antibody titers than healthy males, and females were also a positive factor for antibody titers. Levin EG et al. [32] reported that 6 months after receiving the second dose of COVID-19 vaccines (BNT162b2), NAbs titers were substantially lower among men than women. This may be related to a variety of factors, such as the sex difference caused by smoking [33] and circulating levels of sex steroid hormones [34].

As stated previously, memory B cells are a source of inducible antibodies that provide further protection against infection [35]. The frequency of RBD-specific MBCs was reduced in T2DM patients, which may explain the difference in antibody titers. One point worth our attention is that the percentage of atyMBCs was higher in T2DM patients than in healthy individuals. The function of atyMBCs is still ambiguous, but studies have shown that atyMBCs arose aberrantly in chronic infection, where they displayed impaired antibody-secreting cell differentiation, antiviral effector function, and survival compared with conventional CD27 + memory B cells [36]. Moreover, atyMBCs are dysfunctional in generating specific antibody responses against hepatitis B surface antigen (HBsAg). In chronic hepatitis B (CHB), atyMBCs sacrifice classical memory B cells, and they are likely to impair antigen-specific responses in patients [37]. This might also be one of the reasons why the antibody responses of T2DM patients were compromised after vaccination.

In conclusion, we have reported that inactivated COVID-19 vaccines in T2DM patients were safe and successfully induced the production of anti-RBD-IgG, NAbs, and RBD-specific MBCs. However, the titers and positive rates of antibodies in T2DM patients were obviously lower than those in healthy individuals, as was the frequency of RBD-specific MBCs. mRNA vaccines and inactivated vaccines are two kinds of COVID-19 vaccines widely used. Lee et al. [38] proved that the mRNA vaccine response in patients with T2DM was reassuring. They also found that CoronaVac vaccines had significantly lower levels of anti-RBD IgG antibodies than those who received BNT162b2. However, the conclusion of numerous studies on the antibody responses of mRNA vaccines in the DM population is consistent with our conclusion [12, 13, 20, 39], all suggesting that the antibody titers are significantly lower in the T2DM cohort than in nondiabetic patients. We consider that the main reason is still the dysregulation of immune function in patients with T2DM [40]. The U.S. Centers for Disease Control and Prevention (CDC) recommended that people with T2DM who received the initial series of shots for the Pfizer-BioNTech or Moderna vaccines should get a third shot [41]. In our study, considering that T2DM patients were immunocompromised and antibody titers declined gradually over time, we also recommended that all T2DM patients receive the SARS-CoV-2 vaccination on schedule and might be prioritized to receive vaccines boosters.

Some limitations and possible sources of bias in this study include the following. To begin with, the sample size was relatively small in this study, which possibly hinders some of the results. Then, our study was cross-sectional and could not investigate the immune responses longitudinally in patients with T2DM. Furthermore, antibody titers and memory B-cell levels of participants were not detected in pre-vaccination, nor did we assess the safety after participants received the first dose of vaccines. Finally, we cannot deny the possibility that some data may have been affected by recall bias that came from questionnaires. Given these limitations, more researches in this area are needed to support our conclusions in the future.

## Conclusions

In this cross-sectional study, no serious adverse events were recorded in T2DM patients within 30 days after receiving the inactivated COVID-19 vaccines, and the incidence of adverse events was similar to that in healthy individuals. However, the titers of anti-RBD-IgG and NAbs were significantly reduced in T2DM patients compared to healthy individuals. Antibody titers were correlated with BMI and sex, as well as the days after vaccination. This study also found that the B-cell responses were weakened in T2DM patients following inactivated COVID-19 vaccines. The above conclusions may provide meaningful evidence for medical decision-making and may provide some references for policymakers.

## Supplementary Information

**Additional file 1.** Procedure of RBD-specific memory B cells detection. **Fig. S1.** The positive rates of anti-RBD-IgG and NAbs. (a) All participants of T2DM and HCs (b) BMI in patients with T2DM (c) FPG in patients with T2DM (d) HbA1c in patients with T2DM (e) Treatment with and without insulin in patients with T2DM (f) T2DM alone and T2DM combined with other independent chronic diseases (g) Sex in T2DM and HCs (h) Age in T2DM and HCs (i) Types of vaccines within the T2DM and HCs. **Fig.S2.** The titers of anti-RBD-IgG and NAbs. (a) Sex in T2DM and HCs (b) Age in T2DM and HCs (c)Types of vaccines within the T2DM and HCs. The horizontal dotted lines represent the cutoff values. **Fig.S3.** The gating strategy of flow cytometry for target cells population.

## Data Availability

The datasets used and analyzed during the current study are available from the corresponding author upon reasonable request.

## Abbreviations

T2DM: Type 2 diabetes mellitus
2019-nCoV: 2019 Novel coronavirus
COVID-19: Coronavirus disease 2019
HCs: Healthy controls
Anti-RBD-IgG: Anti-receptor binding domain IgG
NAbs: Neutralizing antibodies
CLIA: Chemiluminescence Immunoassays
RBD-specific MBCs: RBD-specific memory B cells
BMI: Body mass index
HbA1c: Hemoglobin A1C
FPG: Fasting Plasma Glucose
DM: Diabetes mellitus
PBMCs: Peripheral blood mononuclear cells
IQR: Interquartile range
RBD + rMBCs: RBD-specific resting memory B cells
RBD + intMBCs: RBD-specific intermediate memory B cells
RBD + atyMBCs: RBD-specific atypical memory B cells
RBD + actMBCs: RBD-specific activated memory B cells
HBsAg: Hepatitis B surface antigen
CHB: Chronic hepatitis B
CDC: The U.S. Centers for Disease Control and Prevention
