# Supplementary material for: Impaired antibody responses were observed in patients with type 2 diabetes mellitus after receiving the inactivated COVID-19 vaccines
Source: Virol J. 2023 Feb 7;20:22. doi: 10.1186/s12985-023-01983-7 (PMC9902824; doi:10.1186/s12985-023-01983-7)
Supplement: Supplementary file 1 — Additional file 1. Procedure of RBD-specific memory B cells detection. Fig. S1. The positive rates of anti-RBD-IgG and NAbs. (a) All participants of T2DM and HCs (b) BMI in patients with T2DM (c) FPG in patients with T2DM (d) HbA1c in patients with T2DM (e) Treatment with and without insulin in patients with T2DM (f) T2DM alone and T2DM combined with other independent chronic diseases (g) Sex in T2DM and HCs (h) Age in T2DM and HCs (i) Types of vaccines within the T2DM and HCs. Fig.S2. The titers of anti-RBD-IgG and NAbs. (a) Sex in T2DM and HCs (b) Age in T2DM and HCs (c)Types of vaccines within the T2DM and HCs. The horizontal dotted lines represent the cutoff values. Fig.S3. The gating strategy of flow cytometry for target cells population. [file 12985_2023_1983_MOESM1_ESM.docx]

**Additional file 1**

**1 Procedure of RBD-specific memory B cells detection**

To obtain the antigen probe, the biotinylated SARS-CoV-2 spike RBD protein (40592-V08H2-B, Sino Biological, Beijing, China) and streptavidin-BV421 (405225, Biolegend, California, USA) were mixed at a 4:1 molar ratio and then incubated for 1 h at 4 °C. The same volume of PBS (phosphate buffered solution) as the whole blood was added to dilute the blood, and then 3 ml Histopaque (10771, Sigma-Aldrich, St Louis, Missouri, USA) was added to the centrifuge tube. After that, the blood was slowly and carefully added to the upper layer of the Histopaque. The middle layer was absorbed and added to PBS after centrifugation for 30 minutes and then centrifuged at 2500 rpm for 10 minutes. After the supernatant was removed, 200 µl flow staining buffer (FACS, including 2% FBS) was used to resuspend the cells. Finally, antigen probes (1:33.3) and the following fluorescent-coupled antibodies were added to the sample: anti-human CD3 (300430, Biolegend), anti-human CD19 (302212, Biolegend), anti-human CD27 (356406, Biolegend), anti-human CD21 (354918, Biolegend), anti-human IgM (314524, Biolegend), and anti-human IgG Fc (410722, Biolegend). The above antibodies were stained [in the condition of](javascript:;) 4℃ in the dark. The cells were rinsed with FACS after 30 minutes and detected by flow cytometry (CytoFLEX, Beckman Coulter). The data were analyzed by FlowJo software (V10.0.7).

| **(a)** | **(b)**  **** |  |  |
| --- | --- | --- | --- |
| **(c)** | **(d)**   |  |  |
| **(e)**  **** | **(f)**  **** |  |  |
| **(g)**  **** |  |  |  |
| **(h)** |  |  |  |
| **(i)** |  |  |  |

**Fig. S1 The positive rates of** **anti-RBD-IgG and NAb****s.**

**(a)** All [participant](javascript:;)s of T2DM and HCs **(b)** BMI in patients with T2DM **(c)** FPG in patients with T2DM **(d)** HbA1c in patients with T2DM **(e)** Treatment with and without insulin in patients with T2DM **(f)** T2DM alone and T2DM combined with other independent chronic diseases **(g)** Sex in T2DM and HCs **(h)** Age in T2DM and HCs **(i)** Types of vaccines within the T2DM and HCs

| **(a)** |  |
| --- | --- |
| **(b)** |  |
| **(c)** |  |

**Fig. S2** **The titers of anti-RBD-IgG and NAbs****.**

**(a)** Sex in T2DM and HCs **(b)** Age in T2DM and HCs **(c)**Types of vaccines within the T2DM and HCs. The horizontal dotted lines represent the cutoff values.


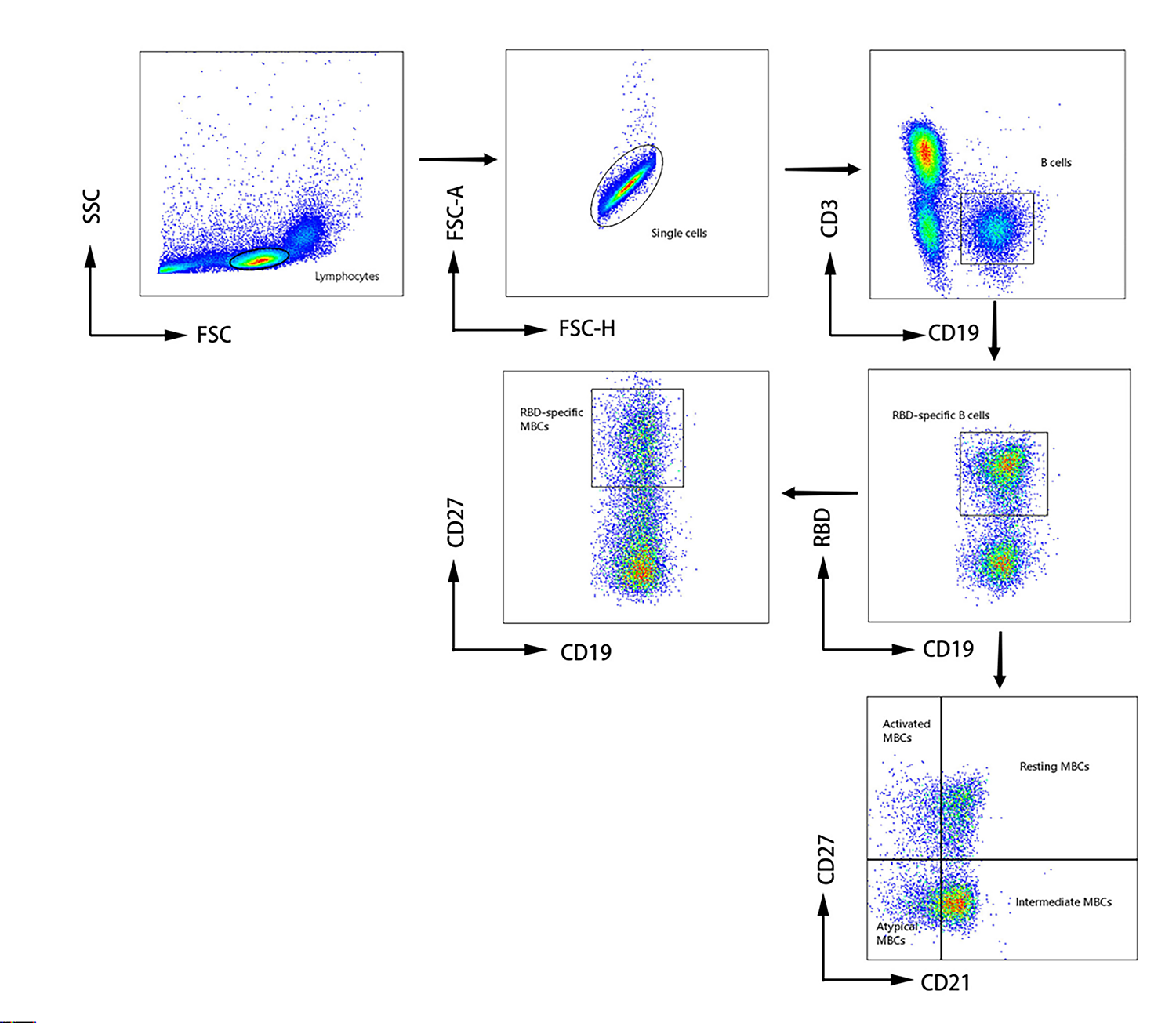


**Fig. S3 The gating strategy of flow cytometry for target cells population.**
